# Insights into the Relationship between Periodontitis and Systemic Sclerosis Based on the New Periodontitis Classification (2018): A Cross-Sectional Study

**DOI:** 10.3390/diagnostics14050540

**Published:** 2024-03-04

**Authors:** Andreea Ciurea, Alina Stanomir, Petra Șurlin, Iulia Cristina Micu, Cristina Pamfil, Daniel Corneliu Leucuța, Simona Rednic, Giulio Rasperini, Andrada Soancă, Adrian Bogdan Țigu, Alexandra Roman, Andrei Picoș, Ada Gabriela Delean

**Affiliations:** 1 Department of Periodontology, Faculty of Dental Medicine, Iuliu Hatieganu University of Medicine and Pharmacy, 400012 Cluj-Napoca, Romania; andreea_candea@yahoo.com (A.C.); alina.stanomir@yahoo.com (A.S.); i.cristina.micu@gmail.com (I.C.M.); andrapopovici@gmail.com (A.S.); 2 Emergency County Clinical Hospital Cluj, 400006 Cluj-Napoca, Romania; cristinapamfil.umfcluj@gmail.com (C.P.); srednic.umfcluj@gmail.com (S.R.); 3 Department of Periodontology, Faculty of Dental Medicine, University of Medicine and Pharmacy Craiova, 200349 Craiova, Romania; surlinpetra@gmail.com; 4 Department of Rheumatology, Faculty of Medicine, Iuliu Hatieganu University of Medicine and Pharmacy, 400006 Cluj-Napoca, Romania; 5 Department of Medical Informatics and Biostatistics, Faculty of Medicine, Iuliu Hatieganu University of Medicine and Pharmacy, 400347 Cluj-Napoca, Romania; danny.ldc@gmail.com; 6 Department of Biomedical, Surgical and Dental Sciences, University of Milan, Foundation IRCCS Ca’ Granda Policlinic, 20122 Milan, Italy; giulio.rasperini@unimi.it; 7 Research Centre for Advanced Medicine (MEDFUTURE), Iuliu Hatieganu University of Medicine and Pharmacy, 400347 Cluj-Napoca, Romania; adrianbogdantigu@gmail.com; 8 Department of Prevention in Dental Medicine, Faculty of Dental Medicine, Iuliu Hatieganu University of Medicine and Pharmacy, 400083 Cluj-Napoca, Romania; 9 Department of Odontology and Endodontics, Faculty of Dental Medicine, Iuliu Hatieganu University of Medicine and Pharmacy, 400001 Cluj-Napoca, Romania; adadelean@yahoo.com

**Keywords:** systemic scleroderma, periodontitis, inflammation, biomarker

## Abstract

(1) Background: This study aimed to assess the periodontitis burden in systemic sclerosis patients and the possible association between them, and the degree to which some potential risk factors and two potential diagnostic biomarkers may account for this association. (2) Methods: This cross-sectional study included a test group (systemic sclerosis patients) and a control group (non-systemic sclerosis patients). Both groups benefited from medical, periodontal examination and saliva sampling to determine the salivary flow rate and two inflammatory biomarkers (calprotectin, psoriasin). A systemic sclerosis severity scale was established. (3) Results: In the studied groups, comparable periodontitis rates of 88.68% and 85.85%, respectively, were identified. There were no significant differences in the severity of periodontitis among different systemic sclerosis severity, or in the positivity for anti-centromere and anti-SCL70 antibodies. Musculoskeletal lesions were significantly more common in stage III/IV periodontitis (*n* = 33, 86.84%) than in those in stage I/II (*n* = 1, 100%, and *n* = 3, 37.5%, respectively) (*p* = 0.007). Comparable levels of the inflammatory mediators were displayed by the two groups. There were no significant differences in calprotectin and psoriasin levels between diffuse and limited forms of systemic sclerosis. (4) Conclusions: Within the limitations of the current study, no associations between systemic sclerosis and periodontitis, or between their risk factors, could be proven.

## 1. Introduction

Systemic sclerosis (SSc) or scleroderma is an immune-mediated rheumatic disease characterized by fibrosis of the skin and internal organs and vasculopathy, exhibiting multisystemic pathologies, clinical heterogeneity, a high mortality rate, and extreme morbidity [1].

The large range of oral manifestations in SSc is frequently overshadowed by systemic conditions [2], complicated care, and deterioration of quality of life in affected patients. Nonetheless, oral findings such as tongue rigidity, thin white lips, microstomia, oro-facial telangiectasia, oral candidiasis, mandibular bone resorption, tooth decay, dental erosions, and periodontitis have been shown to be associated with SSc [2,3].

Periodontitis is a chronic infectious inflammatory disease of the tooth-supporting apparatus frequently reported in the general population [4], as well as in some systemically affected groups [5,6,7,8]. Periodontitis has been epidemiologically associated with various chronic diseases, such as cardiovascular diseases, type 2 diabetes mellitus, certain cancers, inflammatory bowel disease, and rheumatoid arthritis. Emerging evidence indicates that periodontitis treatment ameliorates surrogate markers of comorbid conditions [6,9].

Reported data have indicated a relationship between periodontitis and SSc [2], mostly with the latter’s diffuse form [9]. Twelve case-control studies investigating periodontal conditions and their related risk factors in SSc patients were identified [9,10,11,12,13,14,15,16,17,18,19,20]. Only six studies revealed an association between the two diseases, with SSc being positively correlated with periodontitis [9,13,16,19,20] or periodontitis-related parameters [14]. A very recent meta-analysis including some of the above-mentioned reports highlighted that periodontitis was more common in SSc patients [odds ratio (OR) 7.007; 95% confidence interval (CI) 3.529, 13.915)] and was associated with more changes of clinical parameters such as probing depth (PD) [standard mean difference (SMD) 3.101; 95% CI 1.374, 4.829] and clinical attachment loss (CAL) (SMD 2.584; 95% CI 0.321, 4.846) [2]. The large heterogeneity in characterization of the periodontal state in terms of periodontal parameters [9,10,11,18] and the different or inconsistent periodontitis case definitions [12,13,14,15,16,19,20] make comparisons or estimations of periodontitis frequency in SSc patients difficult, while also complicating the design of coherent prophylactic approaches to efficiently reduce the adverse oral effects of SSc [21].

Attempts have been made to identify common biological markers for the diagnosis, severity, and prognosis of both SSc and periodontitis in order to alert clinicians and construct effective intervention algorithms for SSc patients. Two interesting molecules, calprotectin and psoriasin, have emerged as possible molecular diagnostic tools. Calprotectin (S100A8/A9) is the major cytoplasmic protein in resting and stimulated neutrophils [22], and is also expressed in gingival epithelial cells, activated mononuclear macrophages, vascular endothelial cells [23], and platelets [24]. Calprotectin is involved in multiple cellular processes [22], and has important regulatory effects on immune–inflammatory reactions [23,25]. Due to its zinc binding capacity, calprotectin possesses antimicrobial properties. Direct contact of *Porphyromonas gingivalis* lipopolysaccharide increases calprotectin’s release from neutrophils. Moreover, calprotectin, through its intracellular function, can limit the invasiveness of *Porphyromonas gingivalis* [26,27,28]. Mutations in the calcium-binding loop of S100A9 may determine the loss of calprotectin’s intracellular antimicrobial activity, probably due to the loss of chelating capability of bivalent cations [29].

Increased levels of calprotectin have been shown to be associated with certain inflammatory conditions [25,30]. Calprotectin can be easily measured and used as a candidate biomarker of inflammatory diseases. Fecal calprotectin levels were validated as a biomarker for the diagnosis and longitudinal evaluation of inflammatory bowel disease [30]. In addition, serum calprotectin levels were considered as a prognostic biomarker for the course and outcome of hospitalized COVID-19 patients [31]. It has been shown that increased concentrations of calprotectin in periodontitis-affected patients linked calprotectin to periodontitis [25] and its clinical parameters. In in vivo animal models, periodontal inflammation increased the calprotectin (S100A8/S100A9) expression by gingival and periodontal ligament fibroblasts [32]. Gingival epithelial cells might be another source for the increased gingival crevicular fluid levels of calprotectin in periodontitis patients with diabetes [28]. Calprotectin has been shown to increase the production of cytokines such as IL-6 and IL-8 in human gingival fibroblasts via TLR4-ROS-NF-κB, MAPKs, and S100A9-TLR4 signaling pathways, demonstrating its proinflammatory potential [33,34]. Notably, S100A8 demonstrated heightened expression in immature preosteogenic stromal cells, which diminished as cells became more committed to the pre-osteoblastic stage, which hints at a potential role for S100A8 in osteoblast differentiation. The activation of S100A9 may occur exclusively during the later stages of differentiation [35].

Calprotectin may be considered a marker of inflammation in periodontitis, but other data is necessary to clarify its role [25].

Psoriasin (S100A7) is an antimicrobial protein that acts together with other molecules as an “alarmin” to prime skin cells for the production of immunotropic cytokines, which intensify inflammation. Psoriasin has been shown to be related to different intracellular and extracellular functions, the exacerbation of immune-inflammatory reactions, and osteoclastic activity [36,37]. Salivary psoriasin levels were also found to be associated with the clinical signs of SSc [38] and the development of gingivitis [39] in a dose-dependent manner. Also, elevated levels of psoriasin in saliva and gingival crevicular fluid were found to be associated with periodontal parameters such as increased probing depth and bleeding on probing [40,41,42,43,44]. However, there is a lack of scientific data with respect to psoriasin as a potential diagnostic biomarker in periodontitis.

Against this background, the primary aim of the present study is to investigate the association between SSc and periodontitis and the degree to which some risk factors of these diseases, including two potential diagnostic biomarkers, may account for this association. The null hypothesis tested here is that there are no differences in the levels of these biomarkers between SSc patients and a control group of patients. Another aim of this study is to determine the burden of periodontitis in SSc patients based on currently recognized case definitions. The goal of this is to assess the level of agreement between the 2018 European Federation of Periodontology (EFP)/American Academy of Periodontology (AAP) classification and the former one and to establish the foundation to design uniform periodontal therapeutic algorithms.

## 2. Materials and Methods

### 2.1. Study Design

For this prospective cross-sectional study, patients were recruited from units at two university hospitals: the Rheumatology Clinic of the Emergency County Clinical Hospital Cluj-Napoca (test group) and the Cardiology Department of the Clinical Rehabilitation Hospital Cluj-Napoca (control group). Ethical approval was obtained from these two hospitals (1098/15 January 2020 and 4048/23 April 2021) and from Iuliu Hațieganu University of Medicine and Pharmacy Cluj-Napoca (96/9 March 2020). All participants voluntarily provided written informed consent prior to the clinical examination and biological sample collection. The study complies with the tenets of the Declaration of Helsinki regarding experiments on human subjects. To promote accuracy of the reporting, the paper was written in accordance with the STROBE (Strengthening the Reporting of Observational Studies in Epidemiology) guidelines [45].

This study was performed between March 2020 and December 2022, with some interruptions during COVID lockdowns and peaks in the pandemic. The study group included SSc patients, while the control group included non-SSc patients with cardiovascular pathologies. Both groups benefited from complex medical investigations related to their systemic disorder, a full-mouth periodontal examination provided in a hospital setting, and saliva sampling to determine salivary flow rate and two inflammation-related biomarkers. Medical data were collected from the subjects’ medical records.

### 2.2. Participants, Inclusion Criteria, and Sample Size Calculation

SSc patients included in the test group were recruited consecutively and daily, according to the hospital register. The patients were referred from hospitals located in the north-central part of the country. The patients were enrolled in line with the inclusion criteria: adult patients diagnosed with limited (lcSSc) or diffuse (dcSSc) type of SSc in accordance with recent EULAR/ACR criteria [46]. The exclusion criteria were patients aged <18 years, edentulous patients, severe health conditions due to SSc that prevent oral examination, presence of concurrent autoimmune conditions, acute conditions, antibiotic treatment in the last 6 months, oncological diseases in the last 5 years, recent periodontal treatment (in the last year), and the presence of fewer than six teeth.

The control group was recruited 2 days per week based on a 2:1 ratio in relation to the SSc group, with matching for sex for almost all patients and matching for age of ±5 years. The other inclusion criteria were no history of any autoimmune conditions and the presence of at least six teeth.

The sample size was calculated using GPower 3.1 (Heinrich Heine University, Düsseldorf, Germany) taking into account CAL as the primary outcome. An effect size of 0.50 with α of 0.050, a power level of 80%, a test:control allocation ratio of 2:1, and a two-tailed *p*-value were considered, which resulted in a minimum of 48 enrolled patients in the test group and 96 in the control group [47].

### 2.3. Evaluation of Demographic and Systemic Medical Characteristics

Demographic data, such as age, sex, residential location (urban/rural), education, body mass index (kg/m^2^), and behavioural characteristics (smoking and alcohol consumption), were collected from all patients’ medical records. On the day of the oral examination, the obtained data were checked for completeness and content, and any issues were clarified before the examination.

Patients were divided into smokers/non-smokers/former smokers and alcohol consumers/non-consumers in accordance with the National Center for Health Statistics criteria listed in the Centers for Disease Control (CDC) glossary [48,49].

Blood samples were taken from all subjects included in this study in the morning after a 12-h fast, and standard parameters were measured: basal glycemia, total cholesterol, low-density-lipoprotein cholesterol, high-density-lipoprotein cholesterol, and triglycerides. Signs of systemic involvement, as well as diagnosis of cardiovascular disease and type 1 and 2 diabetes mellitus, based on current guidelines were identified in the medical records.

### 2.4. Systemic Sclerosis-Associated Variables of Interest

Systemic involvement in SSc was divided into eight groups of pathological changes. Each of three groups included several representative systemic conditions and received a maximum impairment score (MIS) based on the number of disorders included in the group. Each patient received a score for each of these three groups depending on the number of diagnosed systemic conditions. Each of the other five groups was represented by a single condition or parameter. The following groups of pathologies and their included systemic conditions were considered in the present study: (1) cardiac involvement score (0 = no pathology, 1 = myocardial fibrosis, 2 = pericarditis, 3 = arrythmias; MIS = 6); (2) digestive involvement score (0 = no pathology, 1 = gastro-esophageal reflux disease—GERD, 2 = dysphagia, 3 = gastroparesis, 4 = malabsorption; MIS = 10); (3) musculoskeletal effects score (0 = no pathology, 1 = arthritis, 2 = tenosynovitis, 3 = acroosteolysis, 4 = myositis, 5 = calcinosis; MIS = 15); (4) interstitial fibrous pneumopathy; (5) telangiectasia; (6) global Rodnan score; (7) anti-centromere antibodies; and (8) anti-SCL70 antibodies.

### 2.5. Periodontal Parameters of Interest and Assessment Method: Case Definitions of Periodontal Conditions

#### 2.5.1. Parameters of Interest and Assessment Method

The examinations were performed by six experienced investigators previously calibrated (A.C., I.C.M., A.S., A.P., A.S., A.G.D.) [7,50] and one young specialist (A.St.). Before the study, they received written instructions on the study design, periodontal evaluation, and data collection protocols. All investigators twice underwent training for performing the examinations under the supervision of two senior periodontists (A.R., P.Ș.). Intra- and inter-examiner reproducibility values were 0.95 and 0.94, respectively.

All patients underwent a full-mouth periodontal examination (excluding wisdom teeth) using standard methodology [50] and equipment (dental mirror, periodontal probe/UNC-15 periodontal probe; Hu-Friedy, Chicago, IL, USA). Each tooth was evaluated in six sites for PD, gingival recession, and CAL, while full-mouth gingival bleeding index (GBI) and oral hygiene index (OHI) scores were calculated in accordance with standard clinical definitions [50,51,52,53]. Tooth mobility and the number of missing teeth were also recorded.

#### 2.5.2. Case Definition of Periodontal Conditions

Periodontitis was diagnosed based on the latest case definition system proposed by the EFP/AAP released in 2018 [54,55]. This diagnosis was based on the presence of interdental CAL at a minimum of two non-adjacent teeth, or buccal/oral CAL ≥ 3 mm associated with PD > 3 mm. The identified periodontitis cases were divided into stages based on the most severe destruction and certain complex elements such as tooth mobility, posterior bite collapse, deep pockets, and furcation lesions [54,55]. A four-level categorical scale was generated: (a) stage I (mild) periodontitis; (b) stage II (moderate) periodontitis; (c) stage III plus IV (severe) periodontitis; and (d) gingivitis and periodontally healthy cases.

A second case definition system (2012 CDC/AAP) was alternatively applied. For this, a four-level categorical scale was established [51]: (a) mild periodontitis, (b) moderate periodontitis, (c) severe periodontitis, and (d) gingivitis plus periodontally healthy cases.

### 2.6. Saliva Sampling and Salivary Flow Rate Measurement

Both unstimulated and stimulated saliva were sampled before the periodontal examination to measure the salivary flow rate and determine two inflammation-related mediators. Briefly, saliva samples were collected during the first hours of the day, in 50 mL sterile centrifuge tubes (STARLAB International GmbH, Hamburg, Germany). Participants were asked to use the passive drooling technique [56] to passively collect saliva in a collection tube immersed in ice for 15 min. All stimulated saliva was obtained by applying 100 µL of 1% citric acid every 30 s for 5 min onto the dorsum of the tongue [57] with a calibrated single-channel pipette (Biohit; Sigma-Aldrich, Darmstadt, Germany). The collection times were recorded.

The saliva samples were immediately frozen at −80 °C until analysis following previously described protocols [58]. The stimulated and unstimulated saliva volumes of the biological samples were determined by weighing the tube before and after collection using an electronic precision scale (FH-2000; Waagenet, Berlin, Germany). The volume was determined by assuming that 1 g of saliva is equivalent to 1 mL. For both unstimulated and stimulated saliva, saliva volume and salivary flow rate calculated by dividing the saliva volume by the sampling time were recorded.

### 2.7. Quantification of Salivary S100A8 and S100A9/Immunoenzymatic Testing (ELISA)

Psoriasin (S100-A7) and Calprotectin L1 (S100-A8/A9) concentrations in the saliva samples were measured. Before testing, saliva samples were thawed, aliquoted, centrifuged for 8 min at 220× *g* and 4 °C, and placed on ice until the loading step. For loading, 100 μL of each sample was plated and further processed in accordance with the manufacturer’s protocol.

Psoriasin (S100-A7) was quantified using an ELISA kit (Cat. No. E1484h; EIAab, Wuhan, China). ELISA sensitivity value of 0.056 ng/mL was considered. Values below the detection limit were replaced with the sensitivity value divided by 2, namely, 0.028 ng/mL.

Calprotectin L1 (S100-A8/A9) concentration was determined using another ELISA kit (Cat. No. ELH-S100A8-9; RayBiotech, Norcross, GA, USA). An ELISA sensitivity value of 0.035 ng/mL was considered. Values below the detection limit were replaced with the sensitivity value divided by 2, namely, 0.0175 ng/mL. For both assays, the absorbance was evaluated at 450 nm using a TECAN SPARK 10M spectrophotometer (Männedorf, Switzerland).

### 2.8. Statistical Analysis

R Environment for Statistical Computing and Graphics (R Foundation for Statistical Computing, Vienna, Austria) version 4.1.2 [R Core Team] was employed for all statistical analyses. We describe qualitative data as the absolute and relative frequencies and quantitative data as medians and interquartile ranges (for skewed distributions). Comparisons between two independent groups for qualitative variables were carried out by Chi-squared or Fisher’s exact test, as needed, while those for quantitative variables were carried out by Wilcoxon’s rank-sum test. A 95% confidence interval computed by bootstrapping for the difference between medians is presented for selected variables. A 0.05 level of significance and two-tailed *p*-values were used for all statistical tests. No corrections for the alpha error inflation rate were performed. The overlap and redistribution of cases calculated using the two case definition systems are illustrated in a Sankey diagram [59]. A series of multiple logistic regression analyses were conducted to estimate the likelihood of advanced periodontitis (stage III/IV) in comparison to early-stage periodontitis (stage II/I) or its absence, and to differentiate between the presence and absence of periodontitis. These models incorporated SSc as a primary independent variable relative to a control group. Additionally, adjustments were made for potential confounders including diabetes, hypertension, and smoking status to ensure the robustness of the associations observed. We evaluated the model’s fit using the Hosmer and Lemeshow test. Additionally, we assessed multicollinearity using the variance inflation factor and correlation analyses. In one model, we adhered to the principle of maintaining a minimum of ten participants for each variable, specifically within the subgroup having the least number of participants for the dependent variable. For another model, constrained by limited degrees of freedom, we constructed a composite confounding score. This score accumulated points for the presence of hypertension, diabetes, and current smoking status, summing these points to avoid model overfitting.

## 3. Results

**Figure 1:**
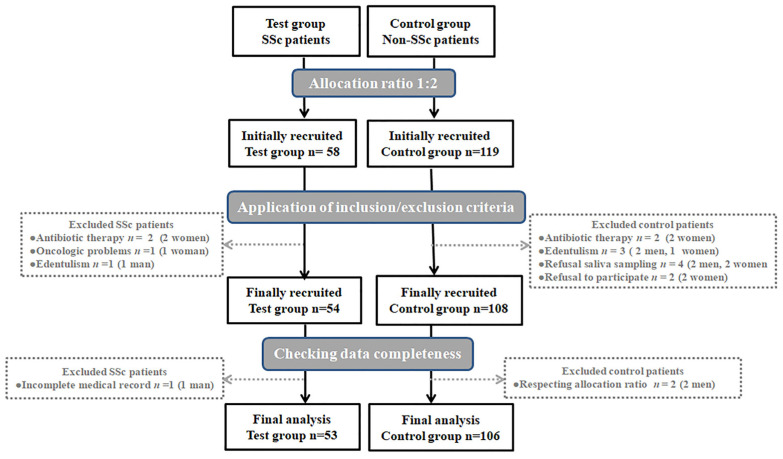
Flow chart of test and control groups. Abbreviations: *n*, number; SSc, systemic sclerosis.

The current study investigated the association between SSc and periodontitis and the degree to which some potential risk factors, including demographical, behavioural, and systemic-related parameters, may account for this association. This study included 53 SSc patients who were age- and sex-matched to 106 non-SSc controls. A flow chart of the patients’ recruitment is presented in Figure 1. The periodontal status was characterized based on the clinical diagnosis according to the two case definition systems, as well as on certain periodontitis-associated surrogate parameters. The periodontitis burden in both groups of patients was also calculated, as well as the agreement of different periodontal conditions diagnosed based on the current and former periodontitis case definition systems.

The characteristics of the patients in both the SSc and control groups based on demographic and behavioral characteristics, as well as general systemic impairments, are provided in Table 1.

Statistically significant differences between the SSc group and controls were identified for residential area (*p* = 0.001), alcohol consumption (*p* = 0.002), education level (*p* < 0.001), diabetes (*p* = 0.002), and hypertension (*p* < 0.001). Cardiac, digestive, and musculoskeletal involvement were described for the SSc group and expressed as scores established based on the number of disorders included in the same category affecting each individual. Most SSc patients had no cardiac disorders (79.25%). Considering cases with digestive and musculoskeletal involvement, the largest proportions of SSc patients belonged to the categories with scores of 1 (30.19%) and 2 (41.51%), respectively. More than half of SSc patients had interstitial pneumopathy, telangiectasia, or anti-SCL antibodies (56.6%, 66.04%, and 75.47%, respectively).

In terms of periodontal conditions based on the current 2018 EFP/AAP case definition system, periodontitis-related and salivary parameters of both SSc and control groups are provided in Table 2. As expected, both unstimulated and stimulated salivary rates in SSc patients were statistically significantly lower than the corresponding values for the control group (both *p* < 0.001). There were no other significant differences in the considered parameters between the two groups. Several multiple logistic regression models were fit to predict stage III/IV vs. stage II/I or no periodontitis, as well as periodontitis diagnosis vs. no periodontitis, in relation to the presence of SSc (vs. control), and adjusted for confounding variables diabetes, hypertension, and smoking status (Supplementary Table S1). No significant relationship was found between the groups.

This study evaluated the impact of infection and inflammation associated with severe periodontal destruction on organ involvement in SSc by analyzing the associative relationships among periodontitis stage categories, organ damage, and specific SSc antibodies. No significant differences were observed among the severity levels of periodontitis in different SSc severity categories in cases with cardiac and digestive involvement, or for positivity for anti-centromere and anti-SCL antibodies (*p* = 0.334, *p* = 0.81, *p* = 1, and *p* = 0.562, respectively). Musculoskeletal lesions were statistically significantly more common in stage III/IV periodontitis patients (*n* = 33, 86.84%) than in stage I or II ones (*n* = 1, 100%, and *n* = 3, 37.5%, respectively) (*p* = 0.007) (Supplementary Material Table S2).

Calprotectin and psoriasin levels were compared between the SSc and control groups in consideration of the periodontal conditions (Table 3). Significantly higher calprotectin levels were observed for non-periodontitis SSc patients than for control patients (*p* = 0.045), and for stage III/IV periodontitis control patients than for the SSc group (*p* = 0.049).

Upon evaluating the levels of inflammatory biomarkers depending on the subtype of SSc, the analysis showed no significant differences in calprotectin and psoriasin levels between diffuse and limited forms of SSc (0.58 ng/mL (−1.42 to 0.31), *p* = 0.174 [n1 = 28, n2 = 21], and 0.26 ng/mL (−0.83 to 1.15), *p* = 0.95 [n1 = 8, n2 = 6], respectively (Supplementary Material Table S3).

Calprotectin and psoriasin levels were not associated with either telangiectasia or cardiac involvement as an expression of SSc severity (*p* = 0.382, *p* = 0.061, and *p* = 0.083, *p* = 0.264, respectively) (Supplementary Material Table S4).

When all of the patients were considered, no correlations of calprotectin levels with age (Spearman’s correlation coefficient 0.08, *p* = 0.312) or GBI score (Spearman’s correlation coefficient 0.08, *p* = 0.324) were identified. Similarly, no correlations of psoriasin levels with age (Spearman’s correlation coefficient −0.01, *p* = 0.926) or GBI score (Spearman’s correlation coefficient −0.06, *p* = 0.446) were identified.

**Figure 2:**
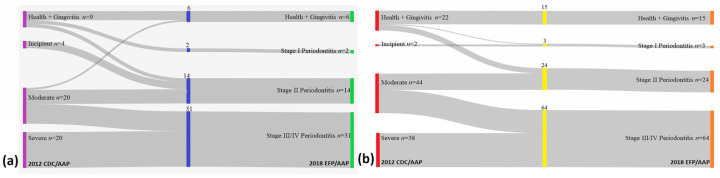
Sankey diagram. (**a**) Systemic sclerosis group. (**b**) Control group. Abbreviations: AAP, American Academy of Periodontology; CDC, Centers for Disease Control; EFP, European Federation of Periodontology; *n*, number.

Reporting periodontal conditions of the study patients by using the two case definition systems (2018 EFP/AAP and 2012 CDC/AAP) revealed a few differences between certain periodontal categories (Table 4, Figure 2). For both SSc and control groups, periodontitis and its severe forms were more frequently diagnosed when the current classification criteria were applied.

## 4. Discussion

The present study investigated the putative association between SSc and periodontitis and the involvement of certain risk factors based on the currently recognized rigorous premises for defining and quantifying these two diseases. This study could not identify any associative relationships between SSc and periodontitis nor between their risk factors. As revealed in Table 2, no differences between the SSc group and controls were identified regarding periodontal diseases (*p* = 0.068), severity-related criteria (*p* = 0.767), periodontitis-associated variables (*p* = 0.932, *p* = 0.706, *p* = 0.720, *p* = 0.462, *p* = 0.628, *p* = 0.925), or potential diagnostic markers (*p* = 0.461, *p* = 0.305). Thus, the null hypothesis could not be rejected.

Based on the new classification criteria, comparable periodontitis rates of 88.68% and 85.85% were detected in the SSc group and controls, respectively, values that are above the mean data for the general population reported in the literature [4]. Moreover, severe periodontitis corresponding to stage III/IV categories affected 71.7% of SSc patients and 66.04% of controls, probably because both diseases profoundly modify the periodontal biotype. Our results are much higher than the mean frequency of 11% of severe periodontitis reported globally [4]. Elsewhere, it has been reported [9] that severe periodontal disease occurred in 39.5% of SSc patients using the 2015 AAP periodontitis case definition combining different parameters and thresholds, which could explain the observed differences as compared with our results. Very recently, a periodontitis frequency of 81.6% in patients with limited SSc was reported [20].

Other studies also reported higher prevalence rates of periodontitis and severe periodontitis cases as well as increased periodontal attachment loss in SSc patients compared to the levels in healthy individuals [2,13,19].

High plaque index scores of both SSc and control groups could explain bleeding index scores that are above the threshold associated with the state of gingival health. Patients from both groups displayed a relatively small number of shallow pockets (PD = 5 mm) and a markedly reduced number of deep pockets (PD ≥ 6 mm), which could have been due to localized periodontal destruction and could explain the relatively low levels of local inflammation. No differences in median PD values were observed between the two groups (*p* = 0.701), which corresponds to other data revealing no statistically significant differences in the mean PD values between SSc patients and controls (2.99 ± 0.59 mm vs. 3.16 ± 0.58, *p* = 0.111) [19].

There are inconsistencies in the reported gingival inflammation indices in SSc patients. Some authors reported significantly higher bleeding on probing values in SSc patients than in controls [11,14] but other studies [9,19] and a recent meta-analysis [2] reported reduced bleeding on probing in SSc, probably consecutive to local fibrosis, which confirms our results.

Comparable levels of the inflammatory mediators calprotectin and psoriasin were displayed by the SSc and control groups (3.12 ng/mL vs. 3.51 ng/mL and 0.67 ng/mL vs. 0.75 ng/mL, respectively). Meanwhile, significantly higher calprotectin levels were observed in periodontitis controls than in periodontitis-SSc patients [3.525 (2.22–4.438) vs. 2.676 (2.04–3.231), *p* = 0.049]. In contrast, non-periodontitis patients with SSc displayed significantly higher calprotectin values than the corresponding controls [4.372 (4.11–4.607) vs. 2.899 (1.669–3.443), *p* = 0.045]. In the SSc group, calprotectin levels decreased from non-periodontitis patients to those at stage III/IV, but in controls, an opposite increasing trend was instead observed. The latter is plausible since increased calprotectin levels were reported in both cardiovascular patients and periodontitis-affected individuals. Moreover, calprotectin has been identified to have potential prognostic value for cardiovascular mortality independently of diabetes status [60]. The components of the heterodimer, S100A8/S100A9, seem to have different salivary trends. It was reported that the concentration of S100A8 in saliva was 1.6 and 1.8 times higher in participants with stage II and stage III–IV periodontitis compared to those without periodontitis. However, there was no significant difference observed in the concentration of S100A9 in saliva (61). Interestingly, lower levels of gingival crevicular fluid of calprotectin (S100A8/S100A9) were observed in stage II and stage III–IV periodontitis patients compared to those with incipient forms or no periodontitis. Moreover, it appears that there are no differences in blood concentrations of S100A8 according to periodontitis status [61]. However, there are inconsistent data on the gingival sulcular and plasmatic calprotectin levels in individuals with periodontitis [62].

Our study did not find any correlation between salivary calprotectin and the GBI score (*p* = 0.324), but others reported that salivary calprotectin concentrations could reflect the degree of periodontal inflammation [63,64]. Moreover, although age-related differences in serum calprotectin concentration have been reported [22], the present study could not confirm this (*p* = 0.324).

In both the SSc and control groups, psoriasin failed to differentiate between mild or healthy periodontal conditions and severe periodontitis (*p* = 0.206 and *p* = 0.706, respectively), which contrasts with previously reported results [37].

Our results should be considered with caution since the controls were non-SSc patients but with systemic diseases, which is considered a limitation of this study. The sampling of healthy controls was impossible in the context of the COVID-19 pandemic and the strict medical restrictions in place.

There are other limitations to our study that should be mentioned. As this was an observational study, causative associations could not be confirmed; moreover, given the nature of this study, there was a risk of confounding bias, since the use of two groups can allow selection biases to influence the results.

Meanwhile, some strengths of this study can be highlighted. The prospective nature of the data collection in our study confers an advantage over retrospective studies. Another positive feature of our study was the detection of inflammatory mediators as putative diagnostic markers in whole saliva, since its sampling is a non-invasive, simple, safe, and stress-free procedure. Few reported studies have determined the levels of the two markers that we utilized in saliva. The double reporting of the periodontal status is another strength of this work as it allows comparison with previous as well as future research.

To the best of our knowledge, this is the first study to define the periodontal conditions in SSc patients based on the 2018 EFP/AAP classification. For the SSc group, 11 patients with moderate periodontitis as defined by the 2012 CDC/AAP classification were considered to have severe periodontal destruction (stage III/IV) according to the 2018 EFP/AAP case definition. In addition, four patients with incipient periodontitis and two patients with healthy/gingivitis conditions as defined by the 2012 CDC/AAP classification were considered as stage II periodontitis by the 2018 EFP/AAP classification. The same tendency to reclassify patients was observed in the control group. Thus, 26 patients with moderate periodontitis as defined by the 2012 CDC/AAP classification were considered as stage III/IV periodontitis cases according to the 2018 EFP/AAP classification. The stage II periodontitis group under the 2018 EFP/AAP classification consisted of 18 patients from the moderate periodontitis group and 6 patients from the healthy/gingivitis group based on the 2012 CDC/AAP classification.

This trend of reclassifying patients has already been reported by our team, with a misclassification rate of 23.41% (*n* = 33) found among a group of stroke patients [7]. In the present study, higher rates of misclassification of periodontal conditions were identified, namely, 39.6% (*n* = 21) for the SSc group and 31.1% (*n* = 33) for the controls.

The obtained results could lay a foundation for the inclusion of oral screening in the regular evaluation of each SSc-diagnosed patient.

With the ongoing development of inflammatory marker assessment and laboratory tools, there are still important challenges related to the identification of appropriate molecular diagnostic biomarkers of both periodontitis and SSc. Further investigations of large groups of SSc patients with an appropriate choice of controls should be carried out.

## 5. Conclusions

No differences were observed between the SSc group and controls concerning periodontal diseases, severity-related criteria, or periodontitis-associated variables. To elucidate the relationship between periodontitis and systemic sclerosis, additional research would be necessary with a broader SSc group and a control group with minimal systemic pathologies.

The present study could not provide a background to sustain calprotectin and psoriasin as possible diagnostic biomarkers of the severity of both SSc and periodontitis. Distinct patterns of calprotectin levels in saliva were observed in our patient groups in relation to the severity of periodontitis. This finding warrants further investigation to elucidate the underlying reasons.

Increased frequencies of periodontitis and its severe forms in both groups of patients highlight the urgent need to implement extensive screening methods in daily clinical practice.

## Figures and Tables

**Table 1:** Patients’ characteristics.

Group	SSc (*n* = 53)	Control (*n* = 106)	*p*-Value
Age (years), median (IQR)	52 (45–59)	53 (46.25–61)	0.544
Sex (female), *n* (%)	44 (83.02)	85 (80.19)	0.667
Residential area (rural), *n* (%)	26 (49.06)	25 (23.58)	**0.001**
Smoking, *n* (%)			0.253
Yes	7 (13.21)	16 (15.09)	
Former smoker	4 (7.55)	2 (1.89)	
No	42 (79.25)	88 (83.02)	
Alcohol consumption, *n* (%)	10 (18.87)	46 (43.4)	**0.002**
Education, *n* (%)			**<0.001**
Primary school	2 (3.77)	0 (0)	
Gymnasium	8 (15.09)	1 (0.94)	
High school	30 (56.6)	75 (70.75)	
University	12 (22.64)	30 (28.3)	
Vocational school	1 (1.89)	0 (0)	
BMI (kg/m^2^), median (IQR)	25.25 (22.32–30.12)	26.52 (23.9–29.32)	0.21
Diabetes, *n* (%)	2 (3.77)	25 (23.58)	**0.002**
Hypertension, *n* (%)	15 (28.3)	72 (67.92)	**<0.001**
Dyslipidemia, *n* (%)	26 (49.06)	57 (53.77)	0.575
Systemic manifestations in SSc group			
Cardiac score, *n* (%)	11 (20.75) *		
0	42 (79.25)		
1	10 (18.87)		
2	1 (1.89)		
Digestive score, *n* (%)	33 (62.26) *		
0	20 (37.74)		
1	16 (30.19)		
2	14 (26.42)		
3	2 (3.77)		
4	1 (1.89)		
Musculoskeletal score, *n* (%)	43 (81.13) *		
0	10 (18.87)		
1	18 (33.96)		
2	22 (41.51)		
3	1 (1.89)		
4	2 (3.77)		
Interstitial fibrous pneumopathy	30 (56.6)		
Telangiectasia	35 (66.04)		
Rodnan score, median (IQR) [range]	7 (3–10) [0–38]		
Anti-centromere antibodies, *n* (%)	19 (35.85)		
Anti-SCL70 antibodies, *n* (%)	40 (75.47)		

Abbreviations: BMI, body mass index; IQR, interquartile range; *n*, number; SCL70, topoisomerase type 1; SSc, Systemic sclerosis; *, scores above 0.

**Table 2:** Periodontal status and salivary parameters of the study groups.

Group	SSc	Control	*p*-Value
	(*n* = 53)	(*n* = 106)	
OHI (%), median (IQR)	36 (25–80)	40.5 (20–71)	0.591
GBI (%), median (IQR)	28 (13–45)	26.5 (12–52)	0.932
No. of examined teeth, median (IQR)	17 (11–23)	17 (11–22.75)	0.856
Periodontal diagnosis, *n* (%)			0.068
Periodontal health	3 (5.66)	14 (13.21)	
Gingivitis	3 (5.66)	1 (0.94)	
Periodontitis	47 (88.68)	91 (85.85)	
Stage, *n* (%)			0.767
Stage I/II	9 (16.98)	21 (19.81)	
Stage III/IV	38 (71.7)	70 (66.04)	
Non-periodontitis	6 (11.32)	15 (14.15)	
Maximum CAL, median (IQR)	6 (5–7)	6 (4–8)	0.706
Median CAL, median (IQR)	2.2 (1.29–3.29)	2.5 (0.82–3.34)	0.72
Minimum CAL, median (IQR)	0 (0–1)	0 (0–1)	0.269
Median PD, median (IQR)	2 (2–3)	2 (2–2.38)	0.701
No. of sites with PD = 5 mm	3 (1–9)	3 (0–8.75)	0.462
No. of sites with PD > 5 mm	3 (1–11)	3 (0–11)	0.628
No. of sites with PD ≥ 6 mm	0 (0–2)	0 (0–2)	0.925
Salivary parameters			
Unstimulated salivary flux (mL/min), median (IQR)	0.22 (0.14–0.35)	0.41 (0.38–0.43)	**<0.001**
Stimulated salivary flux (mL/min), median (IQR)	1.32 (0.6–2.2)	2.43 (2.24–2.64)	**<0.001**
Calprotectin (ng/mL), median (IQR)	3.12 (2.3–4.15)	3.51 (2.32–4.39)	0.461 [n1 = 49, n2 = 98]
Psoriasin (ng/mL), median (IQR)	0.67 (0.19–1.24)	0.75 (0.36–1.49)	0.305 [n1 = 14, n2 = 32]

Abbreviations: CAL, clinical attachment loss; GBI, gingival bleeding index; OHI, oral hygiene index; IQR, interquartile range; *n*, number; n1, n2, number of subjects in the analysis, in case of missing data (bellow the detection limit); PD, probing depth; SSc, systemic sclerosis.

**Table 3:** Intra- and inter-group comparisons between calprotectin and psoriasin levels based on periodontal conditions.

Non-Periodontitis				
Biomarker	SSc (*n* = 6)	Control (*n* = 15)	Difference (95% CI)	*p*-value
Calprotectin (ng/mL), median (IQR)	4.372 (4.11–4.607)	2.899 (1.669–3.443)	1.473 (−2.965–86)	**0.045** [n1 = 6, n2 = 14]
Psoriasin (ng/mL), median (IQR)	0.028 (0.028–0.028)	0.028 (0.028–0.028)	0 (0–0)	0.277 [n1 = 0, n2 = 3]
**Stage I/II Periodontitis**				
Biomarker	SSc (*n* = 9)	Control (*n* = 21)	Difference (95% CI)	*p*-value
Calprotectin (ng/mL), median (IQR)	3.45 (2.588–4.635)	3.358 (1.833–4.263)	0.092 (−1.657–0.879)	0.476 [n1 = 9, n2 = 20]
Psoriasin (ng/mL), median (IQR)	0.028 (0.028–0.36)	0.028 (0.028–0.028)	0 (0–0.11)	0.246 [n1 = 1, n2 = 6]
**Stage III/IV Periodontitis**				
Biomarker	SSc (*n* = 38)	Control (*n* = 70)	Difference (95% CI)	*p*-value
Calprotectin (ng/mL), median (IQR)	2.676 (2.04–3.231)	3.525 (2.22–4.438)	0.848 (0–1.285)	**0.049** [n1 = 34, n2 = 64]
Psoriasin (ng/mL), median (IQR)	0.028 (0.028–0.145)	0.028 (0.028–0.338)	0 (0–0)	0.747 [n1 = 13, n2 = 23]
*p*-value *	0.068	0.305		
*p*-value †	0.206	0.706		

Abbreviations: CI, confidence interval; IQR, interquartile range; *n*, number; n1, n2, number of subjects in the analysis, in case of missing data (bellow the detection limit); SSc, systemic sclerosis; *, intragroup calprotectin modifications; †, intragroup psoriasin modifications.

**Table 4:** The frequency of periodontal conditions based on two case definition systems.

Periodontal Conditions	Frequency *n* (%)			
2012 CDC/AAP System 2018 EFP/AAP System	2012 CDC/AAP		2018 EFP/AAP	
	SSc	Control	SSc	Control
Health + Gingivitis	9 (16.98)	22 (20.75)	6 (11.32)	15 (14.15)
Mild Periodontitis—Stage I	4 (7.54)	2 (1.88)	2 (3.77)	1 (0.94)
Moderate Periodontitis—Stage II	20 (37.73)	44 (41.50)	14 (26.41)	26 (24.52)
Severe Periodontitis—Stage III + IV	20 (37.73)	38 (35.84)	31 (58.49)	64 (60.37)
**Total**	53	106	53	106

Abbreviations: AAP, American Academy of Periodontology; CDC, Center for Disease Control; EFP, European Federation of Periodontology; *n*, number; SSc, systemic sclerosis; data are presented as numbers and percentages.

## Data Availability

The datasets generated during the current study are available from the corresponding author on reasonable request.

## Supplementary Materials

The following supporting information can be downloaded at: https://www.mdpi.com/article/10.3390/diagnostics14050540/s1, Table S1. Multiple logistic regressions predicting stage III/IV or periodontitis, based on group, and adjusted for diabetes, hypertensio, and smoking status; Table S2: Systemic involvement in systemic sclerosis in relation with periodontitis severity; Table S3: Calprotectin and psoriasin levels in relation with the form of systemic sclerosis; Table S4: Calprotectin and psoriasin levels in relation with telangiectasia and cardiac involvement, in systemic sclerosis patients.

## References

[1] Lescoat A., Huang S., Carreira P.E., Siegert E., de Vries-Bouwstra J., Distler J.H.W., Smith V., Del Galdo F., Anic B., Damjanov N. (2023). Cutaneous Manifestations, Clinical Characteristics, and Prognosis of Patients with Systemic Sclerosis Sine Scleroderma: Data from the International EUSTAR Database. JAMA Dermatol..

[2] Zhang S., Zhu J., Zhu Y., Zhang X., Wu R., Li S., Su Y. (2021). Oral Manifestations of Patients with Systemic Sclerosis: A Meta-Analysis for Case-Controlled Studies. BMC Oral Health.

[3] Ciurea A., Rednic N.V., Soancă A., Micu I.C., Stanomir A., Oneț D., Șurlin P., Filipescu I., Roman A., Stratul Ș.I. (2023). Current Perspectives on Periodontitis in Systemic Sclerosis: Associative Relationships, Pathogenic Links, and Best Practices. Diagnostics.

[4] Oral Disorders Collaborators (2020). Global, Regional, and National Levels and Trends in Burden of Oral Conditions from 1990 to 2017: A Systematic Analysis for the Global Burden of Disease 2017 Study. J. Dent. Res..

[5] Wu C.-Z., Yuan Y.-H., Liu H.-H., Li S.-S., Zhang B.-W., Chen W., An Z.-J., Chen S.-Y., Wu Y.-Z., Han B. (2020). Epidemiologic Relationship between Periodontitis and Type 2 Diabetes Mellitus. BMC Oral Health.

[6] Sanz M., Del Castillo A.M., Jepsen S., Gonzalez-Juanatey J.R., D’Aiuto F., Bouchard P., Chapple I., Dietrich T., Gotsman I., Graziani F. (2020). Periodontitis and Cardiovascular Diseases: Consensus Report. J. Clin. Periodontol..

[7] Costea C.A., Christodorescu R., Soancă A., Roman A., Micu I.C., Stratul Ș.I., Rusu D., Popescu D.M., Popa-Wagner A., Bulboacă A.E. (2022). Periodontitis in Ischemic Stroke Patients: Case Definition Challenges of the New Classification Scheme (2018). J. Clin. Med..

[8] Pan W., Wang Q., Chen Q. (2019). The Cytokine Network Involved in the Host Immune Response to Periodontitis. Int. J. Oral Sci..

[9] da Silva G.S.G., de Melo M.L.M., Leão J.C., Carvalho A.T., Porter S., Duarte A.L.B.P., Dantas A.T., Gueiros L.A. (2019). Oral Features of Systemic Sclerosis: A Case-Control Study. Oral Dis..

[10] Wood R.E., Lee P. (1988). Analysis of the Oral Manifestations of Systemic Sclerosis (Scleroderma). Oral Surg. Oral Med. Oral Pathol..

[11] Leung W.K., Chu C.H., Mok M.Y., Yeung K.W.S., Ng S.K.S. (2011). Periodontal Status of Adults with Systemic Sclerosis: Case-Control Study. J. Periodontol..

[12] Chu C.H., Yeung C.M.K., Lai I.A., Leung W.K., Mok M.Y. (2011). Oral Health of Chinese People with Systemic Sclerosis. Clin. Oral. Investig..

[13] Baron M., Hudson M., Tatibouet S., Steele R., Lo E., Gravel S., Gyger G., El Sayegh T., Pope J., Fontaine A. (2014). The Canadian Systemic Sclerosis Oral Health Study: Orofacial Manifestations and Oral Health-Related Quality of Life in Systemic Sclerosis Compared with the General Population. Rheumatology.

[14] Elimelech R., Mayer Y., Braun-Moscovici Y., Machtei E.E., Balbir-Gurman A. (2015). Periodontal Conditions and Tumor Necrosis Factor-Alpha Level in Gingival Crevicular Fluid of Scleroderma Patients. Isr. Med. Assoc. J..

[15] Iordache C., Antohe M.-E., Chirieac R., Ancuța E., Țănculescu O., Ancuța C. (2019). Volumetric Cone Beam Computed Tomography for the Assessment of Oral Manifestations in Systemic Sclerosis: Data from an EUSTAR Cohort. J. Clin. Med..

[16] Isola G., Williams R.C., Lo Gullo A., Ramaglia L., Matarese M., Iorio-Siciliano V., Cosio C., Matarese G. (2017). Risk Association between Scleroderma Disease Characteristics, Periodontitis, and Tooth Loss. Clin. Rheumatol..

[17] Isola G., Palazzo G., Polizzi A., Murabito P., Giuffrida C., Lo Gullo A. (2021). Association of Systemic Sclerosis and Periodontitis with Vitamin D Levels. Nutrients.

[18] Mayer Y., Elimelech R., Balbir-Gurman A., Braun-Moscovici Y., Machtei E.E. (2013). Periodontal Condition of Patients with Autoimmune Diseases and the Effect of Anti-Tumor Necrosis Factor-α Therapy. J. Periodontol..

[19] Pischon N., Hoedke D., Kurth S., Lee P., Dommisch H., Steinbrecher A., Pischon T., Burmester G.R., Buttgereit F., Detert J. (2016). Increased Periodontal Attachment Loss in Patients with Systemic Sclerosis. J. Periodontol..

[20] Jud P., Wimmer G., Meinitzer A., Strohmaier H., Schwantzer G., Moazedi-Fürst F., Schweiger L., Brodmann M., Hafner F., Arefnia B. (2023). Periodontal Disease and Its Association to Endothelial Dysfunction and Clinical Changes in Limited Systemic Sclerosis: A Case-Control Study. J. Periodontal Res..

[21] Almeida T.G., Ferreira A.R.H., da Silva F.S., Chaves C.C., Assunção B.N., Martins P.S., das Dores A.S., de Moura R.M.F., Andrade J.A., Santos F.P.S.T. (2023). Oral Health Education for Systemic Sclerosis Patients: A Booklet Report. PEC Innov..

[22] Kotsiou O.S., Papagiannis D., Papadopoulou R., Gourgoulianis K.I. (2021). Calprotectin in Lung Diseases. Int. J. Mol. Sci..

[23] Wang S., Song R., Wang Z., Jing Z., Wang S., Ma J. (2018). S100A8/A9 in Inflammation. Front. Immunol..

[24] Gao H., Xu J., He L., Meng H., Hou J. (2021). Calprotectin Levels in Gingival Crevicular Fluid and Serum of Patients with Chronic Pe-Riodontitis and Type 2 Diabetes Mellitus before and after Initial Periodontal Therapy. J. Periodontal Res..

[25] Wei L., Liu M., Xiong H. (2019). Role of Calprotectin as a Biomarker in Periodontal Disease. Mediat. Inflamm..

[26] Johnstone K.F., Herzberg M.C. (2022). Antimicrobial Peptides: Defending the Mucosal Epithelial Barrier. Front. Oral Health.

[27] Kido J.-I., Kido R., Kataoka M., Fagerhol M.K., Nagata T. (2003). Calprotectin Release from Human Neutrophils Is Induced by Porphyromonas Gingivalis Lipopolysaccharide via the CD-14–Toll-like Receptor–Nuclear Factor κB Pathway. J. Periodontal Res..

[28] Hiroshima Y., Sakamoto E., Yoshida K., Abe K., Naruishi K., Yamamoto T., Shinohara Y., Kido J.-I., Geczy C.L. (2018). Advanced Glycation End-Products and Porphyromonas Gingivalis Lipopolysaccharide Increase Calprotectin Expression in Human Gingival Epithelial Cells. J. Cell. Biochem..

[29] Champaiboon C., Sappington K.J., Guenther B.D., Ross K.F., Herzberg M.C. (2009). Calprotectin S100A9 Calcium-Binding Loops I and II Are Essential for Keratinocyte Resistance to Bacterial Invasion. J. Biol. Chem..

[30] Jukic A., Bakiri L., Wagner E.F., Tilg H., Adolph T.E. (2021). Calprotectin: From Biomarker to Biological Function. Gut.

[31] Silvin A., Chapuis N., Dunsmore G., Goubet A.-G., Dubuisson A., Derosa L., Almire C., Hénon C., Kosmider O., Droin N. (2020). Elevated Calprotectin and Abnormal Myeloid Cell Subsets Discriminate Severe from Mild COVID-19. Cell.

[32] Gao H.Y., Huang B.X., Hou J.X., Meng H.X. (2020). Preliminary study on the expression and distribution of S100A8 and S100A9 in healthy and experimental periodontitis tissues. Zhonghua Kou Qiang Yi Xue Za Zhi.

[33] Gao H., Hou J., Meng H., Zhang X., Zheng Y., Peng L. (2017). Proinflammatory Effects and Mechanisms of Calprotectin on Human Gingival Fibroblasts. J. Periodontal Res..

[34] Zheng Y., Hou J., Peng L., Zhang X., Jia L., Wang X., Wei S., Meng H. (2014). The Pro-Apoptotic and pro-Inflammatory Effects of Calprotectin on Human Periodontal Ligament Cells. PLoS ONE.

[35] Zreiqat H., Howlett C.R., Gronthos S., Hume D., Geczy C.L. (2007). S100A8/S100A9 and Their Association with Cartilage and Bone. J. Mol. Histol..

[36] Charoenpong H., Osathanon T., Pavasant P., Limjeerajarus N., Keawprachum B., Limjeerajarus C.N., Cheewinthamrongrod V., Palaga T., Lertchirakarn V., Ritprajak P. (2019). Mechanical Stress Induced S100A7 Expression in Human Dental Pulp Cells to Augment Osteoclast Differentiation. Oral Dis..

[37] Takahashi T., Asano Y., Yamashita T., Nakamura K., Saigusa R., Miura S., Ichimura Y., Toyama T., Hirabayashi M., Taniguchi T. (2018). A Potential Contribution of Psoriasin to Vascular and Epithelial Abnormalities and Inflammation in Systemic Sclerosis. J. Eur. Acad. Dermatol. Venereol..

[38] Giusti L., Sernissi F., Donadio E., Ciregia F., Giacomelli C., Giannaccini G., Mazzoni M.R., Lucacchini A., Bazzichi L. (2016). Salivary Psoriasin (S100A7) Correlates with Diffusion Capacity of Carbon Monoxide in a Large Cohort of Systemic Sclerosis Patients. J. Transl. Med..

[39] Dommisch H., Skora P., Hirschfeld J., Olk G., Hildebrandt L., Jepsen S. (2019). The Guardians of the Periodontium-Sequential and Differential Expression of Antimicrobial Peptides during Gingival Inflammation. Results from in Vivo and in Vitro Studies. J. Clin. Periodontol..

[40] Dommisch H., Staufenbiel I., Schulze K., Stiesch M., Winkel A., Fimmers R., Dommisch J., Jepsen S., Miosge N., Adam K. (2015). Expression of Antimicrobial Peptides and Interleukin-8 during Early Stages of Inflammation: An Experimental Gingivitis Study. J. Periodontal Res..

[41] Eberhard J., Pietschmann R., Falk W., Jepsen S., Dommisch H. (2009). The Immune Response of Oral Epithelial Cells Induced by Single-species and Complex Naturally Formed Biofilms. Oral Microbiol. Immunol..

[42] Kido J., Nakamura T., Kido R., Ohishi K., Yamauchi N., Kataoka M., Nagata T. (1999). Calprotectin in Gingival Crevicular Fluid Correlates with Clinical and Biochemical Markers of Periodontal Disease. J. Clin. Periodontol..

[43] Kido J., Bando M., Hiroshima Y., Iwasaka H., Yamada K., Ohgami N., Nambu T., Kataoka M., Yamamoto T., Shinohara Y. (2012). Analysis of Proteins in Human Gingival Crevicular Fluid by Mass Spectrometry: Mass Spectrometric Analysis of Gingival Crevicular Fluid. J. Periodontal Res..

[44] Nakamura T., Kido J.-I., Kido R., Ohishi K., Yamauchi N., Kataoka M., Nagata T. (2000). The Association of Calprotectin Level in Gingival Crevicular Fluid with Gingival Index and the Activities of Collagenase and Aspartate Aminotransferase in Adult Periodontitis Patients. J. Periodontol..

[45] von Elm E., Altman D.G., Egger M., Pocock S.J., Gøtzsche P.C., Vandenbroucke J.P., STROBE Initiative (2014). The Strengthening the Reporting of Observational Studies in Epidemiology (STROBE) Statement: Guidelines for Reporting Observational Studies. Int. J. Surg..

[46] Van Den Hoogen F., Khanna D., Fransen J. (2013). Classification Criteria for Systemic Sclerosis: An American College of Rheumatology/European League Against Rheumatism Collaborative Initiative: ACR/EULAR Classification Criteria for SSc. Arthritis Rheum..

[47] Faul F., Erdfelder E., Lang A.-G., Buchner A. (2007). G*Power 3: A Flexible Statistical Power Analysis Program for the Social, Behavioral, and Biomedical Sciences. Behav. Res. Methods.

[48] (2019). NHIS—Adult Tobacco Use—Glossary. https://www.cdc.gov/nchs/nhis/tobacco/tobacco_glossary.htm.

[49] (2019). NHIS—Adult Alcohol Use—Glossary. https://www.cdc.gov/nchs/nhis/alcohol/alcohol_glossary.htm.

[50] Micu I.C., Roman A., Ticala F., Soanca A., Ciurea A., Objelean A., Iancu M., Muresan D., Caracostea G.V. (2020). Relationship between Preterm Birth and Post-Partum Periodontal Maternal Status: A Hospital-Based Romanian Study. Arch. Gynecol. Obstet..

[51] Eke P.I., Page R.C., Wei L., Thornton-Evans G., Genco R.J. (2012). Update of the Case Definitions for Population-based Surveillance of Periodontitis. J. Periodontol..

[52] Ainamo J., Bay I. (1975). Problems and Proposals for Recording Gingivitis and Plaque. Int. Dent. J..

[53] Oleary T.J., Drake R.B., Naylor J.E. (1972). The Plaque Control Record. J. Periodontol..

[54] Papapanou P.N., Sanz M., Buduneli N. (2018). Periodontitis: Consensus Report of Workgroup 2 of the 2017 World Workshop on the Classification of Periodontal and Peri-Implant Diseases and Conditions: Classification and Case Definitions for Periodontitis. J. Clin. Periodontol..

[55] Chapple I.L.C., Mealey B.L., Van Dyke T.E., Bartold P.M., Dommisch H., Eickholz P., Geisinger M.L., Genco R.J., Glogauer M., Goldstein M. (2018). Periodontal Health and Gingival Diseases and Conditions on an Intact and a Reduced Periodontium: Consensus Report of Workgroup 1 of the 2017 World Workshop on the Classification of Periodontal and Peri-Implant Diseases and Conditions. J. Periodontol..

[56] Bellagambi F.G., Lomonaco T., Salvo P., Vivaldi F., Hangouët M., Ghimenti S., Biagini D., Di Francesco F., Fuoco R., Errachid A. (2020). Saliva Sampling: Methods and Devices. An Overview. Trends Analyt. Chem..

[57] Sacon M.B., Esteves C.V., Florezi G.P., Gonçalves A.F., Pannuti C.M., Lemos Junior C.A. (2018). Comparison of Two Methods for Sialometry: Weighing and Volume Techniques. Clin. Lab. Res. Dent..

[58] Henson B.S., Wong D.T. (2010). Collection, Storage, and Processing of Saliva Samples for Downstream Molecular Applications. Methods Mol. Biol..

[59] SankeyMATIC Sankeymatic.com. https://sankeymatic.com/.

[60] Løfblad L., Hov G.G., Åsberg A., Videm V. (2021). Inflammatory Markers and Risk of Cardiovascular Mortality in Relation to Diabetes Status in the HUNT Study. Sci. Rep..

[61] Kim H.-D., Karna S., Shin Y., Vu H., Cho H.-J., Kim S. (2021). S100A8 and S100A9 in Saliva, Blood and Gingival Crevicular Fluid for Screening Established Periodontitis: A Cross-Sectional Study. BMC Oral Health.

[62] Kajiura Y., Lew J.-H., Ikuta T., Nishikawa Y., Kido J.-I., Nagata T., Naruishi K. (2017). Clinical Significance of GCF sIL-6R and Calprotectin to Evaluate the Periodontal Inflammation. Ann. Clin. Biochem..

[63] Zhou M., Meng H.X., Zhao Y.B., Chen Z.B. (2012). Changes of Four Proinflammatory Proteins in Whole Saliva during Experimental Gingivitis. Chin. J. Dent. Res..

[64] Holmström S.B., Lira-Junior R., Zwicker S., Majster M., Gustafsson A., Åkerman S., Klinge B., Svensson M., Boström E.A. (2019). MMP-12 and S100s in Saliva Reflect Different Aspects of Periodontal Inflammation. Cytokine.

