# Supplementary material for: Insights into the Relationship between Periodontitis and Systemic Sclerosis Based on the New Periodontitis Classification (2018): A Cross-Sectional Study
Source: Diagnostics (Basel). 2024 Mar 4;14(5):540. doi: 10.3390/diagnostics14050540 (PMC10931108; doi:10.3390/diagnostics14050540)
Supplement: Supplementary file 1 [file diagnostics-14-00540-s001.zip › diagnostics-2851668-supplementary.pdf]

**Supporting material - Insights into the relationship between periodontitis and systemic sclerosis based on the new periodontitis classification (2018): A cross-sectional study**

**Supplementary Table S1. Multiple logistic regressions predicting stage III/IV or periodontitis, based on group, and adjusted for diabetes, hypertensio, and smoking status.**

| | **OR adjusted** | **(95% CI)** | **p** |
|---|---|---|---|
| **Predict Stage III/IV vs Stage II/1 or no *** | | | |
| Group (SSc vs. Control) | 1.62 | (0.73 - 3.7) | 0.243 |
| Diabetes | 1.01 | (0.4 - 2.65) | 0.984 |
| Hypertension | 1.68 | (0.81 - 3.53) | 0.169 |
| Smoker (former smoker vs. non-smoker) | 1.04 | (0.18 - 8.03) | 0.964 |
| Smoker (actual smoker vs. non-smoker) | 1.46 | (0.55 - 4.37) | 0.467 |
| **Predict Periodontitis (Yes vs. No), model 1** | | | |
| Group (SSc vs. Control) | 2.06 | (0.7 - 6.74) | 0.205 |
| Diabetes | 1.38 | (0.39 - 6.59) | 0.643 |
| Hypertension | 2.72 | (0.99 - 7.85) | 0.055 |
| Smoker (former smoker vs. non-smoker) | 1.02 | (0.14 - 20.98) | 0.986 |
| Smoker (actual smoker vs. non-smoker) | 1.93 | (0.48 - 13.05) | 0.41 |
| **Predict Periodontitis (Yes vs. No), model 2 *** | | | |
| Group (SSc vs. Control) | 2.02 | (0.7 - 6.55) | 0.212 |
| Confounding score | 2.14 | (1.03 - 4.78) | 0.051 |

SSc, systemic sclerosis; OR, odds ratio; CI, confidence interval; Confounding score is the sum of points for having diabetes, hypertension, or being actual smoker; *, models without overfitting.

Supporting material - **Insights into the relationship between periodontitis and systemic sclerosis based on the new periodontitis classification (2018): A cross-sectional study**

Table S2. Systemic involvement in systemic sclerosis in relation with periodontitis severity

| Stage<br>Systemic involvement | Stage I (n=1) | Stage II (n=8) | Stage III/IV (n=38) | *p*-value |
|---|---|---|---|---|
| Cardiac (Yes), n (%) | 0 (0) | 3 (37.5) | 6 (15.79) | 0.334 |
| Digestive (Yes), n (%) | 1 (100) | 4 (50) | 24 (63.16) | 0.81 |
| Musculoskeletal | 1 (100) | 3 (37.5) | 33 (86.84) | **0.007** |
| Anticentromere antibodies (A/B) (Yes), n (%) | 0 (0) | 3 (37.5) | 15 (39.47) | 1 |
| Anti SCL70 antibodies (Yes), n (%) | 1 (100) | 5 (62.5) | 29 (76.32) | 0.562 |

Abbreviations: n, number; SCL70, topoisomerase type 1;

Table S3. Calprotectin and psoriasin levels in relation with the form of systemic sclerosis

| Systemic sclerosis form | Diffuse (n=28) | Limited (n=21) | Difference (95% CI) | *p*-value |
|---|---|---|---|---|
| Calprotectin (ng/mL), median (IQR) | 2.74 (2.32-3.56) | 3.32 (2.19 - 4.63) | 0.58 (-1.42 - 0.31) | 0.174 ●[n1=28,n2=21] |
| Psoriasin (ng/mL), median (IQR) | 0.49 (0.21 - 1.06) | 0.74 (0.29 - 1.24) | 0.26 (-0.83 - 1.15) | 0.95 ●[n1=8,n2=6] |

Abbreviations: CI, confidence interval; IQR, interquartile range; n, number;

Table S4. Calprotectin and psoriasin levels in relation with telangiectasia and cardiac involvement, in systemic sclerosis patients

| Telangiectasia | Yes (n=35) | No (n=18) | Difference (95% CI) | *p*-value |
|---|---|---|---|---|
| Calprotectin (ng/mL), median (IQR) | 2.611 (1.896 – 4.339) | 3.157 (2.413 – 4.030) | 0.546 (-1.134 – 0.497) | 0.382•[n1=35,n2=18] |
| Psoriasin (pg/mL), median (IQR) | 0.028 (0.028 – 0.21) | 0.028 (0.028 – 0.028) | 0 (0 - 0) | 0.061•[n1=35,n2=18] |
| **Cardiac involvement** | **Yes (n=11)** | **No (n=42)** | **Difference (95% CI)** | ***p*-value** |
| Calprotectin (ng/mL), median (IQR) | 4.215 (2.691 – 4.825) | 2.744 (2.197 – 3.417) | 1.471 (-0.064 – 2.128) | 0.083•[n1=11,n2=42] |
| Psoriasin (pg/mL), median (IQR) | 0.028 (0.028 – 0.028) | 0.028 (0.028 – 0.067) | 0 (0 - 0) | 0.264•[n1=11,n2=42] |

Abbreviations: CI, confidence interval; IQR, interquartile range; n, number;
